# Role of optical coherence tomography angiography in Vogt-Koyanagi-Harada disease

**DOI:** 10.3205/oc000179

**Published:** 2021-03-12

**Authors:** Stefano Erba, Andrea Govetto, Antonio Scialdone, Giuseppe Casalino

**Affiliations:** 1 Oftalmico Hospital, ASST Fatebenefratelli Sacco, Milano, Italy

**Keywords:** optical coherence tomography angiography, retinal imaging, Vogt-Koyanagi-Harada disease

## Abstract

Vogt-Koyanagi-Harada disease (VKH) is an autoimmune severe multisystem condition characterized by both ocular and systemic findings that should be promptly recognized and treated. Although invasive imaging modalities, namely fluorescein angiography and indocyanine green angiography, are still the gold standard for the diagnosis and follow-up of the ocular findings in VKH, the role of retinal non-invasive imaging including optical coherence tomography angiography (OCTA) is under investigation and is not mentioned in the current diagnostic criteria of VKH. The aim of this manuscript was to report the clinical course and the multimodal retinal imaging of a VKH case and to discuss the role of OCTA in this condition. Our case supports the evidence that OCTA is able to help determine disease activity and progression in VKH. We therefore contend that OCTA should be considered for future developing diagnostic criteria of this condition.

## Introduction

Vogt-Koyanagi-Harada disease (VKH) is an autoimmune severe multisystem condition characterized by both ocular and systemic findings mediated by an autoimmune attack against melanocytes by T lymphocytes [1].

A bilateral chronic, diffuse granulomatous panuveitis represents the most commonly observed ocular presentation. VKH should be promptly recognized and treated with high-dose systemic steroids in order to avoid progression to sight-threatening complications such as choroidal neovascularization, subretinal fibrosis and late stage of disease, including choroidal depigmentation and sunset glow fundus [1], [2].

Over the last decade, retinal imaging has expanded from fluorescein angiography (FA) and indocyanine green angiography (ICGA) to the so-called multimodal imaging, which includes a wide range of non-invasive imaging modalities such as optical coherence tomography (OCT) and most recently OCT angiography (OCTA). Although combined FA and ICGA are still the gold standard for the diagnosis and follow-up of the ocular findings in VKH [3], non-invasive multimodal imaging has gained a crucial role in the practice of retinal specialists across the world. OCTA provides a non-invasive, dye-free and depth-resolved examination of the retinochoroidal layers by combining structural analysis and vascular flow information [4].

Here we present the clinical course and the multimodal retinal imaging of a VKH case, and we discuss the role of OCTA in this condition.

## Case description

A 24-year-old Asian man presented to our emergency department with a two-day history of blurred vision in both eyes, malaise, photophobia, vertigo, headache, and mild hearing loss. Best-corrected visual acuity (BCVA) was 20/200 in the right eye (RE) and 20/32 in the left eye (LE). Extraocular motility, intraocular pressure and pupillary light reflexes were within normal limits. The patient’s past medical history was unremarkable; he was afebrile, and systemic blood pressure was 115/65 mm Hg. No signs of meningism or neck stiffness were noted.

**Figure 1:**
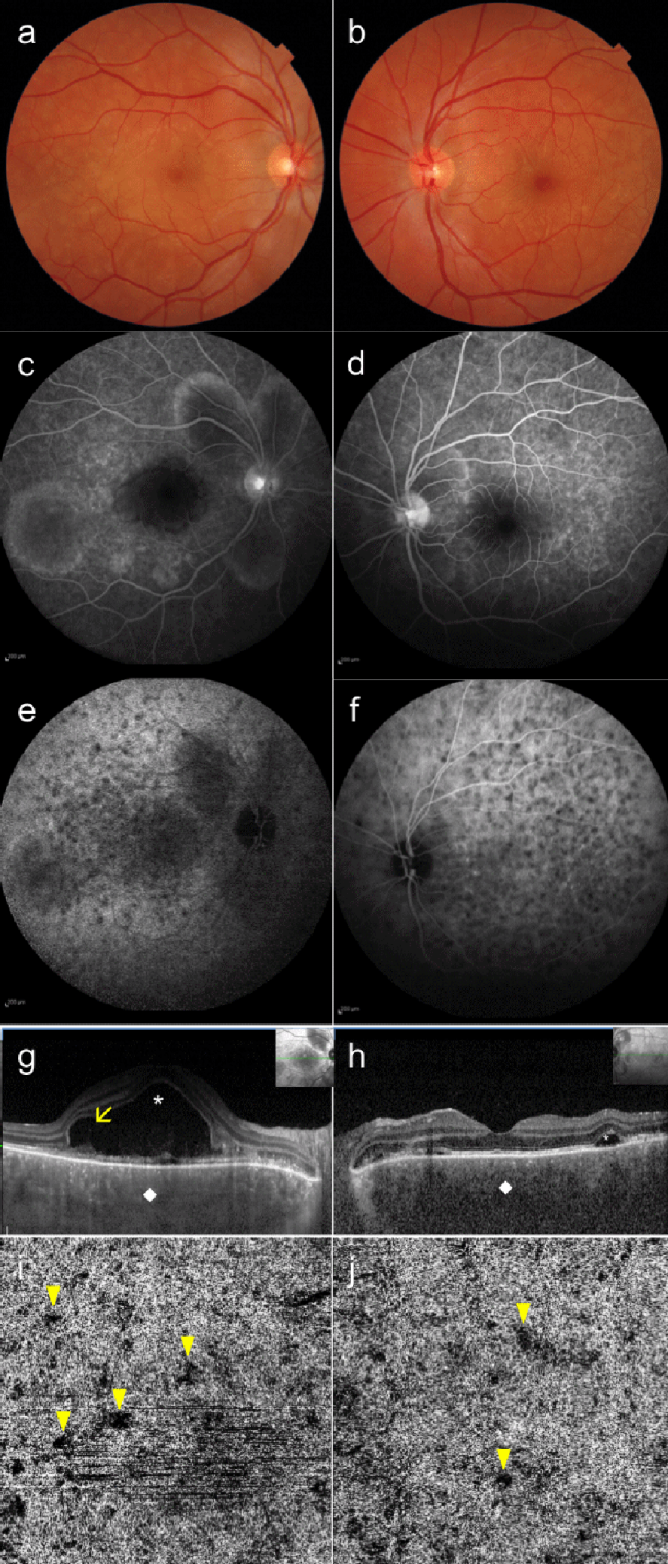
Multimodal retinal imaging of Vogt-Koyanagi-Harada disease of both eyes at presentation. (a, b) Color fundus photograph shows multifocal subretinal detachments (SRD) involving the posterior pole of the right eye and the peripapillary area of both eyes. (c, d) Fluorescein angiography late frames show areas of pinpoint hyperfluorescent leaks of the retinal pigment epithelium with multifocal dye pooling underneath the SRD. (e, f) Indocyanine green angiography (ICGA) frames show persistent multiple hypofluorescent lesions, better seen in the late phases of the examination. (g) Enhanced-depth imaging optical coherence tomography (EDI-OCT) scan of the right eye shows increased subfoveal choroidal thickness (712 µm, white rhombus) and a bacillary detachment (white asterisk) associated with fibrin-like subretinal hyperreflective material (yellow arrow). (h) EDI-OCT of the left eye shows increased subfoveal choroidal thickness (750 µm, white rhombus) and extrafoveal subretinal accumulation of fluid (white asterisk). (i, j) OCT angiography of the choriocapillaris (4.5x4.5 mm) of both eyes shows multiple flow void spots (yellow arrowheads) at the posterior pole that correlate with the hypofluorescent lesions detected on ICGA.

Ocular examination revealed 2+ cells in the anterior chamber of the RE, 1+ cells in the anterior chamber of the LE, 1+ (bio-score) of inflammation in the vitreous cavity of both eyes (BE), bilateral optic nerve head hyperemia and multiple serous retinal detachments (SRD) in BE. Multimodal retinal imaging was performed (Figure 1 (Fig. 1)); FA showed multiple hyperfluorescent retinal pigment epithelium leaks in BE with dye pooling beneath SRD in the RE and normal retinal perfusion with no signs of vasculitis in either eye; ICGA showed persistent multiple hypofluorescent lesions in BE; OCT of the RE showed subfoveal serous macular detachment splitting of the photoreceptors’ layers (also referred to as bacillary detachment) [5]. OCTA (Topcon DRI OCT Triton Plus, Tokyo, Japan) of the choriocapillaris of BE showed multiple areas of decreased flow that correlated with the ICGA hypofluorescent lesions. These imaging findings along with the clinical history were suggestive of VKH in the acute phase [1], [2].

**Figure 2:**
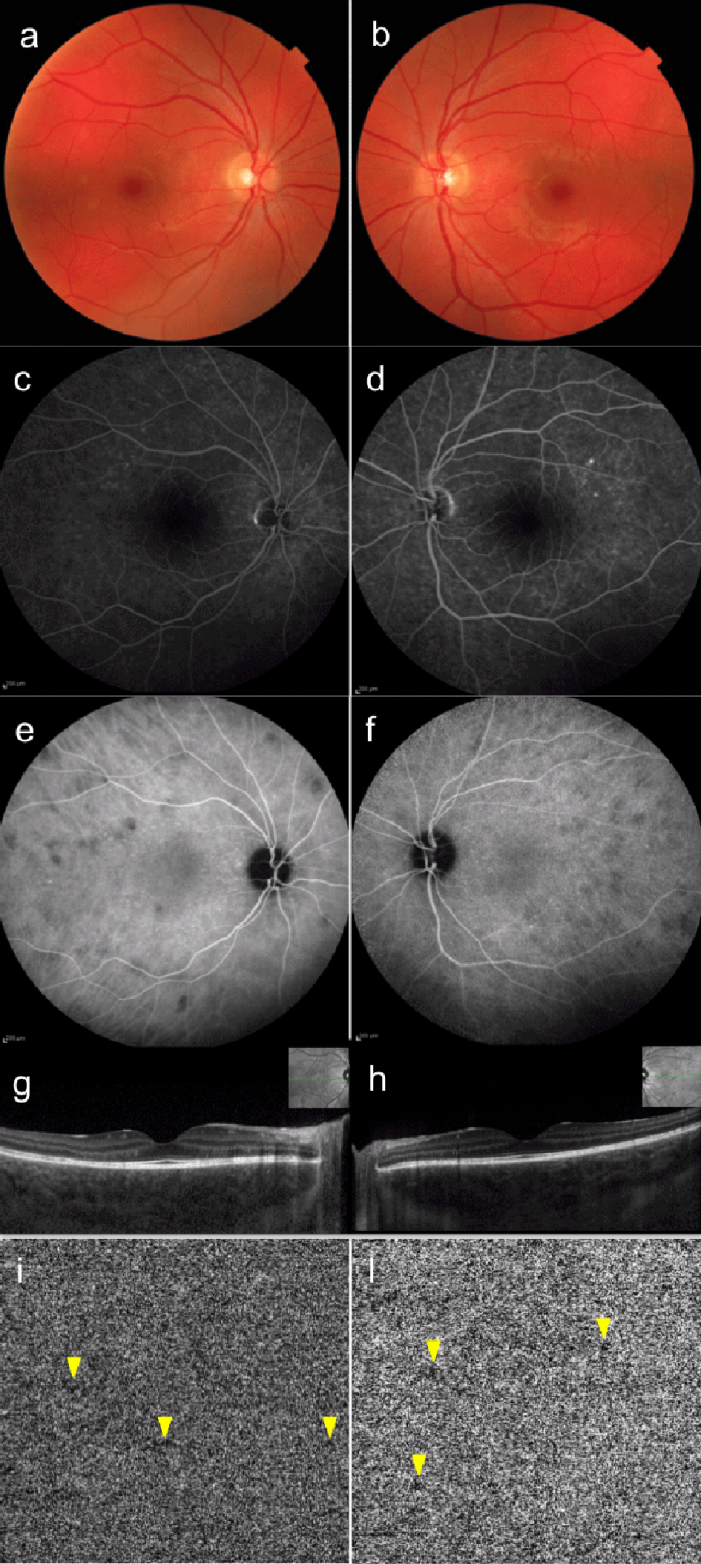
Multimodal non-invasive retinal imaging of Vogt-Koyanagi-Harada disease of both eyes 3 months after presentation. (a, b) Color fundus photograph shows resolution of the multifocal subretinal detachments in both eyes. (c, d) Fluorescein angiography shows residual mild retinal pigment epithelium changes in both eyes. (e, f) Indocyanine green angiography (ICGA) frames show remarkable decrease in size and number of the hypofluorescent lesions visible at presentation. (g, h) Enhanced-depth imaging optical coherence tomography (EDI-OCT) scan of both eyes show regression of the increased subfoveal choroidal thickness (538 µm in the right eye and 548 µm in the left eye) and resolution of the intra- and subretinal exudation. (i, j) OCT angiography of the choriocapillaris (4.5x4.5 mm) of both eyes shows an overall improvement of the choriocapillaris flow with remarkable decrease in size and number of the flow void spots detected at presentation.

The patient was started on immunosuppressive therapy consisting of a tapering course of oral prednisolone starting from 60 mg per day. The patient was closely observed, and during follow-up the presenting symptoms and BCVA steadily improved. Common infectious causes of posterior uveitis were excluded by performing extensive work-up including tuberculosis quantiFERON assay and Treponema serology that resulted negative. Therefore a steroid-sparing agent (cyclosporine A 100 mg twice a day) was given to prevent relapses of the disease. Three months after presentation, BCVA improved to 20/20 in both eyes. OCT revealed complete resolution of the retinal serous detachments and regression of the increased choroidal thickness. ICGA showed marked reduction in size and number of the hypofluorescent lesions with correspondent decrease of the OCTA flow void spots detected at presentation (Figure 2 (Fig. 2)). No signs of relapses were observed during 12 months of follow-up.

## Discussion

VKH may affect neurological and integumentary systems that are critical for the definition of “complete”, “incomplete” or “probable” VKH [6]. According to the Revised Diagnostic Criteria of VKH [6], our case was defined as “incomplete” VKH in view of the absence of the integumentary findings. The consensus on the Revised Diagnostic Criteria (RDC) for VKH has been developed at the first international workshop on VKH in Los Angeles in 1999 and subsequently published in 2001 [6]. In the VKH RDC, OCT is not mentioned, since at that time this technology had not been fully developed, and therefore was not commonly used in the clinical practice [7]. Nowadays, high-resolution OCT technology is a standard of care in ophthalmology [7], and non-invasive imaging modalities including OCTA represent essential tools in the daily retinal clinical practice. Therefore, their role in the diagnosis and monitoring of VKH needs to be defined.

A new set of diagnostic criteria for VKH has been recently proposed [8] and did take into account enhanced depth imaging (EDI) OCT or swept-source OCT (SS-OCT) which are able to image the full-thickness of the choroid [9], [10].

EDI-OCT and SS-OCT in VKH have been shown to reveal loss of focal hyperreflectivity in the inner choroid in both acute and convalescent stages [11] and regression of the increased choroidal thickness after treatment [12], thus representing a candidate biomarker for the degree of choroidal inflammation in the acute phase of the disease and for the response to treatment [12].

Ocular involvement in VKH is characterized by a non-necrotizing diffuse granulomatous inflammatory process of the uvea. Histopathologic studies have shown that the inflammation primarily affects the choroidal stroma with diffuse infiltration of inflammatory cells [13], [14] that may correlate with the hypofluorescent lesions visible on ICGA [3]. However, involvement of choriocapillaris is controversial. Indeed histopathologic studies indicate that choriocapillaris is spared in the acute phase, whereas it may be involved in the degenerative process of the chronic recurrent stage [13], [14].

A recent breakthrough in the field of non-invasive multimodal imaging has been the development of OCTA, which non-invasively provides detailed assessment of the retinal and choroidal vasculature by detecting motions of erythrocytes and visualising blood flow using serial OCT B-scans [4]. OCTA allows for a stratification of different retinal and choroidal layers, which is not possible with FA and ICGA [4]. Therefore, integration of OCTA with the other imaging modalities may offer an opportunity for the understanding of VKH pathophysiology.

Recently, case reports and case series have imaged VKH with OCTA [15], [16], [17], [18].

In a recent case series, Aggarwal et al. [15] reported foci of choriocapillaris flow void on OCTA in the acute stage of the disease, possibly indicating choriocapillaris hypoperfusion. Of note, these foci correlated with ICGA and decreased in number and size after initiation of therapy [15]. Similar OCTA findings in VKH have been reported by another case series [16] and two case reports [17], [18].

Luo et al. [19] identified two OCTA patterns in the choriocapillaris of the acute stage of VKH (one characterized as multiple dark spots of flow void, and the other as highly reflective areas surrounded by light spots with an increased flow area), possibly reflecting different presentation and severity of the condition clinical spectrum. Moreover, they found significant decrease of the choriocapillaris perfusion in the convalescent stage of disease [19].

Our case is in line with the findings reported by Aggarwal et al. [15] as we found choriocapillaris flow void in the acute stage, which substantially improved after treatment in the convalescent stage of the disease.

Taken together, these observations may indicate a possible role of the OCTA findings to determine the disease activity and progression in VKH.

Given the discrepancy between the OCTA and the histopathologic findings regarding the choriocapillaris involvement in the acute phase of VKH, an alternative hypothesis is that the flow void seen on OCTA might reflect a shadowing effect from the overlying subretinal fluid and pigment epithelial detachment [16]. Indeed possible artifacts, difficulty imaging of the deeper choroid and of areas outside the posterior pole represent main limitations of OCTA [15], [16], whose interpretation should therefore be taken with caution and coupled with that of the other imaging techniques.

## Conclusions

Our case supports the evidence that OCTA is able to help determine disease activity and progression of VKH. Although the OCTA limitations and the limited number of cases currently reported do not allow to draw clear pathogenetic insights in VKH, the role of OCTA should be considered for developing future diagnostic criteria of this condition.

## Notes

### Competing interests

The authors declare that they have no competing interests.

### Informed consent

Written informed consent has been obtained from the patient for the publication of this case report.

## References

[1] O’Keefe GAD, Rao NA (2017). Vogt-Koyanagi-Harada disease. Surv Ophthalmol.

[2] Yang P, Ye Z, Xu J, Du L, Zhou Q, Qi J, Liang L, Wang C, Zhou C, Cao Q, Wu L, Kijlstra A (2019). Macular Abnormalities in Vogt-Koyanagi-Harada Disease. Ocul Immunol Inflamm.

[3] Pichi F, Aggarwal K, Neri P, Salvetti P, Lembo A, Nucci P, Gemmy Cheung CM, Gupta V (2018). Choroidal biomarkers. Indian J Ophthalmol.

[4] Spaide RF, Fujimoto JG, Waheed NK, Sadda SR, Staurenghi G (2018). Optical coherence tomography angiography. Prog Retin Eye Res.

[5] Agarwal A, Freund KB, Kumar A, Aggarwal K, Sharma D, Katoch D, Bansal R, Gupta V, OCTA Study Group (2020). Bacillary Layer Detachment in Acute Vogt-Koyanagi-Harada Disease – A Novel Swept-Source Optical Coherence Tomography Analysis. Retina.

[6] Read RW, Holland GN, Rao NA, Tabbara KF, Ohno S, Arellanes-Garcia L, Pivetti-Pezzi P, Tessler HH, Usui M (2001). Revised diagnostic criteria for Vogt-Koyanagi-Harada disease: report of an international committee on nomenclature. Am J Ophthalmol.

[7] Fujimoto J, Swanson E (2016). The Development, Commercialization, and Impact of Optical Coherence Tomography. Invest Ophthalmol Vis Sci.

[8] Yang P, Zhong Y, Du L, Chi W, Chen L, Zhang R, Zhang M, Wang H, Lu H, Yang L, Zhuang W, Yang Y, Xing L, Feng L, Jiang Z, Zhang X, Wang Y, Zhong H, Jiang L, Zhao C, Li F, Cao S, Liu X, Chen X, Shi Y, Zhao W, Kijlstra A (2018). Development and Evaluation of Diagnostic Criteria for Vogt-Koyanagi-Harada Disease. JAMA Ophthalmol.

[9] Spaide RF, Koizumi H, Pozzoni MC (2008). Enhanced depth imaging spectral-domain optical coherence tomography. Am J Ophthalmol.

[10] Chee SP, Chan SN, Jap A (2017). Comparison of Enhanced Depth Imaging and Swept Source Optical Coherence Tomography in Assessment of Choroidal Thickness in Vogt-Koyanagi-Harada Disease. Ocul Immunol Inflamm.

[11] Fong AH, Li KK, Wong D (2011). Choroidal evaluation using enhanced depth imaging spectral-domain optical coherence tomography in Vogt-Koyanagi-Harada disease. Retina.

[12] Nakayama M, Keino H, Okada AA, Watanabe T, Taki W, Inoue M, Hirakata A (2012). Enhanced depth imaging optical coherence tomography of the choroid in Vogt-Koyanagi-Harada disease. Retina.

[13] Inomata H, Rao NA (2001). Depigmented atrophic lesions in sunset glow fundi of Vogt-Koyanagi-Harada disease. Am J Ophthalmol.

[14] Rao NA (2007). Pathology of Vogt-Koyanagi-Harada disease. Int Ophthalmol.

[15] Aggarwal K, Agarwal A, Mahajan S, Invernizzi A, Mandadi SKR, Singh R, Bansal R, Dogra MR, Gupta V, OCTA Study Group (2018). The Role of Optical Coherence Tomography Angiography in the Diagnosis and Management of Acute Vogt-Koyanagi-Harada Disease. Ocul Immunol Inflamm.

[16] Aggarwal K, Agarwal A, Deokar A, Mahajan S, Singh R, Bansal R, Sharma A, Dogra MR, Gupta V, OCTA Study Group (2017). Distinguishing features of acute Vogt-Koyanagi-Harada disease and acute central serous chorioretinopathy on optical coherence tomography angiography and en face optical coherence tomography imaging. J Ophthalmic Inflamm Infect.

[17] Giannakouras P, Andreanos K, Giavi B, Diagourtas A (2017). Optical Coherence Tomography Angiography: Employing a Novel Technique for Investigation in Vogt-Koyanagi-Harada Disease. Case Rep Ophthalmol.

[18] Wintergerst MWM, Herrmann P, Finger RP (2018). Optical Coherence Tomography Angiography for Evaluation of Sattler’s Layer in Vogt-Koyanagi-Harada Disease. Ophthalmic Surg Lasers Imaging Retina.

[19] Luo K, Cai H, Hu Y, Jin C, Gan X, Deng Y, Lu M, Chen H, Su L, Chen G, Chi W (2020). Distinguishing Microvasculature Features of Vogt-Koyanagi-Harada in Patients in Acute and Convalescent Phases Using Optical Coherence Tomography Angiography. Ocul Immunol Inflamm.

